# The Spatial Associations of Cerebral Blood Flow and Spontaneous Brain Activities with White Matter Hyperintensities—An Exploratory Study Using Multimodal Magnetic Resonance Imaging

**DOI:** 10.3389/fneur.2017.00593

**Published:** 2017-11-09

**Authors:** Lin Shi, Xinyuan Miao, Wutao Lou, Kai Liu, Jill Abrigo, Adrian Wong, Winnie C. W. Chu, Defeng Wang, Vincent C. T. Mok

**Affiliations:** ^1^Department of Medicine and Therapeutics, The Chinese University of Hong Kong, Hong Kong, Hong Kong; ^2^Chow Yuk Ho Center of Innovative Technology for Medicine, The Chinese University of Hong Kong, Hong Kong, Hong Kong; ^3^Therese Pei Fong Chow Research Centre for Prevention of Dementia, The Chinese University of Hong Kong, Hong Kong, Hong Kong; ^4^Department of Imaging and Interventional Radiology, The Chinese University of Hong Kong, Hong Kong, Hong Kong

**Keywords:** white matter hyperintensities, cerebral blood flow, functional magnetic resonance imaging, aging, functional connectivity

## Abstract

White matter hyperintensities (WMHs) have been reported to be correlated with functional brain changes, but the association of the specific WMHs distribution pattern with regional functional changes remains uncertain. The aim of this study is to explore the possible spatial correlation of WMH with changes in cerebral blood flow (CBF) and spontaneous brain activities in elderly using a novel approach. The WMHs, CBF, and spontaneous brain activities measured by intrinsic connectivity contrast (ICC), were quantified using multimodal magnetic resonance imaging for 69 elderly subjects. Such approach enables us to expand our search for newly identified correlated areas by drawing strengths of different modes and provides a means for triangulation as well as complementary insights. The results showed significant positive correlations between WMH volumes in the right superior corona radiata and CBF in the left supplementary motor area, as well as between WMH volumes in left anterior limb internal capsule and CBF in the right putamen. Significant correlations of regional WMH volumes and ICC were also detected between the right anterior corona radiata and the left cuneus, and the right superior occipital cortex, as well as between the right superior corona radiata and the left superior occipital cortex. These findings may suggest a regional compensatory functional enhancement accounting for the maintenance of cognitively normal status, which can be supported by the widely observed phenomenon that mild to moderate WMH load could have little effect on global cognitive performance.

## Introduction

With age, the brain undergoes both structural and functional changes (1, 2), among which cerebral white matter changes (WMC) can be widely observed in elderly individuals (3) as white matter hyperintensities (WMHs) on the T2-weighted fluid-attenuated inversion recovery (FLAIR) magnetic resonance imaging (MRI) scans. Periventricular and deep WMC could have various sizes with irregular boundaries. Although the pathology of WMH is not conclusive, it is considered as one factor that caused brain parenchyma damages (4) through demyelination and axonal disruptions. According to the studies across the last two decades, WMHs were suggested to be associated with impaired mobility, mood disorders, and reduced cognitive functions of various domains as measured by standard neuropsychological tests (4), and the WMH severity was reported to be a strong predictor of cognitive decline and vascular dementia (5).

As WMHs are considered as an important manifestation of the cerebral small-vessel disease (6) and have been suggested to be results of cerebral ischemia (7, 8) and neurovascular dysfunction (9), the hemodynamic measures such as cerebral blood flow (CBF) and blood oxygen level dependent (BOLD) fMRI could contribute to the understanding of the mechanism of the damages caused by WMC (10). Arterial spin labeling (ASL) is an MR perfusion technique using arterial water protons as endogenous tracer, which could quantitatively measure the CBF non-invasively. ASL was used in some studies to investigate the effect of WMC on CBF. It has been shown that the CBF values in the WMHs areas were significantly lower than those in normal appearing white matter (10). The decreased CBF was considered a result of the neurodegeneration. Besides CBF, resting-state BOLD fMRI has also been used as a neuroimaging tool in investigating the functional effects of WMC in elderly individuals (11). Intrinsic connectivity contrast (ICC) is a new whole-brain voxel-wise analysis method that reflects the number of functional connections between each voxel and the rest of the brain (12). Altered ICC was reported in diseased and aging populations (13). The functional effect of WMH by simultaneously analyzing multimodal MRI is largely under-studied.

The total volume of WMHs is the major severity measure that has been used in previous studies of WMC, while fewer studies have investigated the importance of WMC localizations. Though some work (14) claimed that the total volume of WMHs might be a good predictor of cognitive decline, emerging recent evidences confirmed that the spatial distribution of WMHs could be associated with changes in specific cognitive domains (15). In particular, Smith et al. (16) employed voxel-wise analysis and reported that the specific WMHs loci might be correlated with impaired executive functions and episodic memory, independent of total WMHs volume. Periventricular WMHs might be associated with cognitive decline (4), whereas the deep WMHs tend to be related to mood disorders like depression (17). The WMHs regions that were related to different cognitive domains varied among studies (4). Despite the studies revealing specific functional changes of regional WMHs, the mechanism of these functional changes induced by WMHs is yet to be clarified and confirmed by imaging evidences.

The importance of examining function activities together with the rather limited scope of previous studies are key motivations for conducting this study. First, WMC could lead to demyelination and axonal disruptions, these structural connection disruptions could in turn cause functional changes in the associated regions, even if the regional WMHs and gray matter areas are remote from each other. Second, most of the previous studies on localization of WMHs focused mainly on associating the total WMH volume or WMHs in some defined WM ROI with gray matter measurements (4). In this study, we aim to investigate the regional correlations between the functional measures and the regional WMH load using an exploratory and exhaustive approach. To achieve this goal, a novel multimodal MRI analysis method was proposed to search for any statistically significant regional correlations between WMH load and functional measures like CBF and ICC. We hypothesized that WMH volume of some specific regions, independent of the total volume, would be associated with regional alternations of blood flow and intrinsic brain activities in cognitively healthy elderly.

## Materials and Methods

### Study Design and Participants

Seventy healthy elderly subjects without clinical stroke, brain tumors, infarcts, psychiatric conditions, and/or risk of Alzheimer’s disease were recruited. The details of inclusion and exclusion criteria were presented in the previous study (18). One subject was excluded due to the incompletion of data acquisition. The remaining 69 subjects were analyzed in this study (Table 1). The study was approved by the Joint Chinese University of Hong Kong-New Territories East Cluster Clinical Research Ethics Committee (CUHK-NTEC CREC) following the ethical standards and procedural requirements described in the Hospital Authority Guide on Research Ethics and the Standard Operating Procedure of the CUHK-NTEC CREC. Formal written consent was obtained from all the participants.

**Table 1:** Population characteristics of the subjects in this study.

Characteristic	Study cohort (*N* = 69)
Age (mean ± SD, years)	70.78 ± 3.94
Gender (female/male)	45/24
Education (mean ± SD, years)	9.06 ± 4.3
Education-adjusted MoCA score	23.26 ± 3.1
Smoking, *n* (%)	8 (11.59%)
Alcohol consumption, *n* (%)	7 (10.14%)
Hypertension, *n* (%)	53 (76.81%)
Family history of stroke, *n* (%)	21 (30.43%)
History of heart disease, *n* (%)	6 (8.7%)

### Procedures

A Hong Kong version of the Montreal Cognitive Assessment (MoCA) was taken as a brief cognitive screen for mild cognitive impairment (18). We followed the standardized procedures in the research clinics at the Prince of Wales Hospital in Hong Kong to collect the MoCA data. The total score of MoCA was calculated with education adjustment.

All subjects were scanned using a 3.0-T scanner (Achieva 3.0T TX Series, Philips Medical System, Best, the Netherlands). Structural MRI scanning was performed using sagittal FLAIR and 3D T1-weighted Turbo Field Echo (TFE). The parameters of FLAIR were: TR = 8,000 ms, TE = 331 ms, TI = 2,400 ms, matrix size = 528 × 528, slice thickness = 1.1 mm with 0.55 mm slice gap, voxel size = 0.44 mm × 0.44 mm, and number of slices = 327. The parameters of T1 were: voxel size = 1.1 mm × 1.1 mm × 0.6 mm isotropic, TR = 7.5 ms, TE = 3.5 ms, flip angle = 8°, matrix size = 240 × 240 × 305. During the resting-state fMRI data acquisition, the subjects were instructed to stay awake with their eyes open and focus on a cross. The acquisition parameters of the BOLD-EPI sequence were: TE/TR = 25/2,050 ms, matrix size = 64 × 64, 47 slices with thickness = 3.2 mm, voxel size = 3.2 mm × 3.2 mm. The total acquisition time for the resting scan was 7 min 10 s. The parameters of the pseudo-continuous ASL sequence were: TE/TR = 14/4,000 ms, label duration = 1,650 ms and post-labeling delay = 1,525 ms, in-plane matrix = 80 × 80, FOV =240 mm × 240 mm, 17 slices with thickness = 7 mm. Twenty pairs of control and label images were acquired for each subject using this sequence.

### WMH Segmentation and Distribution Analysis

The processing of images were performed with Matlab (Mathworks, Natick, MA, USA) and SPM 12.^1^ The WMH were detected by a coarse-to-fine in-house developed mathematical morphology method (19) using the FLAIR and T1-weighted images. The binary mask of WMHs of each subject was coregistrated with the CBF and ICC maps and normalized to the MNI space. To obtain the WMH distribution frequency map, we averaged the normalized WMHs masks across subjects. The WMHs volume was quantified as the sum of volume derived from the binary WHMs mask.

### Calculation of rCBF Maps

Preprocessing steps of the ASL data included slice-timing correction, head motion correction with six head motion parameters, spatial normalization to the MNI space with a resampling resolution of 3 mm × 3 mm × 3 mm, and spatial smoothing with a 6-mm Gaussian kernel. The control and label pCASL images of each subject were realigned with the first control image for rigid-body head motion correction. Afterward, the averaged surround subtraction of control and labeled (Δ*M*) images were derived, and the equilibrium magnetization image *M*_0_ was achieved. The Δ*M* and *M*_0_ images were coregistered to the individual T1W image for further CBF quantification. The quantitative CBF image was estimated based on a single compartment kinetic model (20):

$$\text{CBF}=\frac{6,000\cdot \lambda \cdot \Delta M\cdot e^{\frac{\text{PLD}}{T_{1,\text{blood}}}}}{2\cdot \alpha \cdot T_{1,\text{blood}}\cdot M_{0}\cdot \left(1-e^{\frac{\tau }{T_{1,\text{blood}}}}\right)},$$

where λ = 0.9 is the blood–brain partition coefficient, α = 0.85 is the labeling efficiency, *T*_1,blood_ = 1.65 s is the longitudinal relaxation time of blood, PLD is the post-label delay, and τ is the label duration. The relative CBF maps were then calculated by dividing the mean CBF value of the whole brain for following processing.

### Calculation of ICC Maps

The resting-state fMRI data were preprocessed using the following steps: removal of the first 8 volumes to make the signals reach equilibrium, slice-timing correction, head motion correction with six head motion parameters, coregistration between T1-weighted image and fMRI, T1-weighted image segmentation, spatial normalization to MNI space, spatial smoothing with a 6-mm Gaussian kernel, and band-pass filtering of (0.01–0.08 Hz). The WM and CSF signals were regressed out separately by the PCA approach with 25 components to remove potential non-neuronal noises, and then the images were used for ICC analysis. The calculation of ICC maps was performed by using the CONN toolbox.^2^ At a given voxel, the correlations of the signal at that voxel with that of all other voxels in the brain were calculated, and ICC of that voxel was calculated as the number of correlations that were above a prior given threshold [0.3 was taken in this study as used in our previous study (21)].

### White Matter and Gray Matter Parcelation

Since the aim is to investigate exhaustively the correlations of regional GM measurements (CBF and ICC maps) and WMH volumes, quantitative regional measures need to be generated for parcellated regions using a gray matter atlas for the CBF and ICC maps and a white matter atlas for the WMHs volumes. In this study, AAL atlas^3^ was used for parcellating gray matter, and the DTI atlas^4^ was chosen to parcellate white matter. The vector of WMH volume in a given WM region in the DTI atlas was formed by concatenating the WMHs volumes of that WM region for all subjects. The CBF/ICC values of each AAL region ROI were obtained similarly.

### Statistical Analysis

The partial Pearson correlation of each WMH region with rCBF and ICC regions across subjects was calculated to form two matrices:

R_rCBF(i,j)=corr((rCBF(i),WMH volume(j)),covariates),

R_ICC(i,j)=corr((ICC (i),WMH volume(j)),covariates),

where *i* stands for the region index in the AAL atlas for rCBF or ICC maps, and *j* represents the region index of the DTI atlas for WMH. *Covariates* included age, sex, education-adjusted MoCA score, and total WMH volume.

## Results

**Figure 1:**
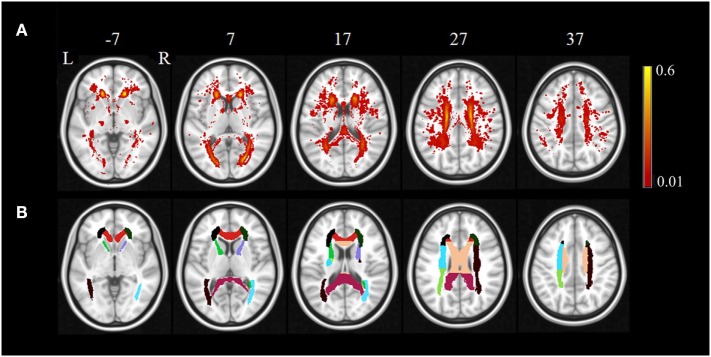
The regional distribution of WMH among all the subjects **(A)** and retained DTI ROIs used in further correlation analysis **(B)**. The color bar in **(A)** indicates the probability of having white matter hyperintensities in each voxel.

**Figure 2:**
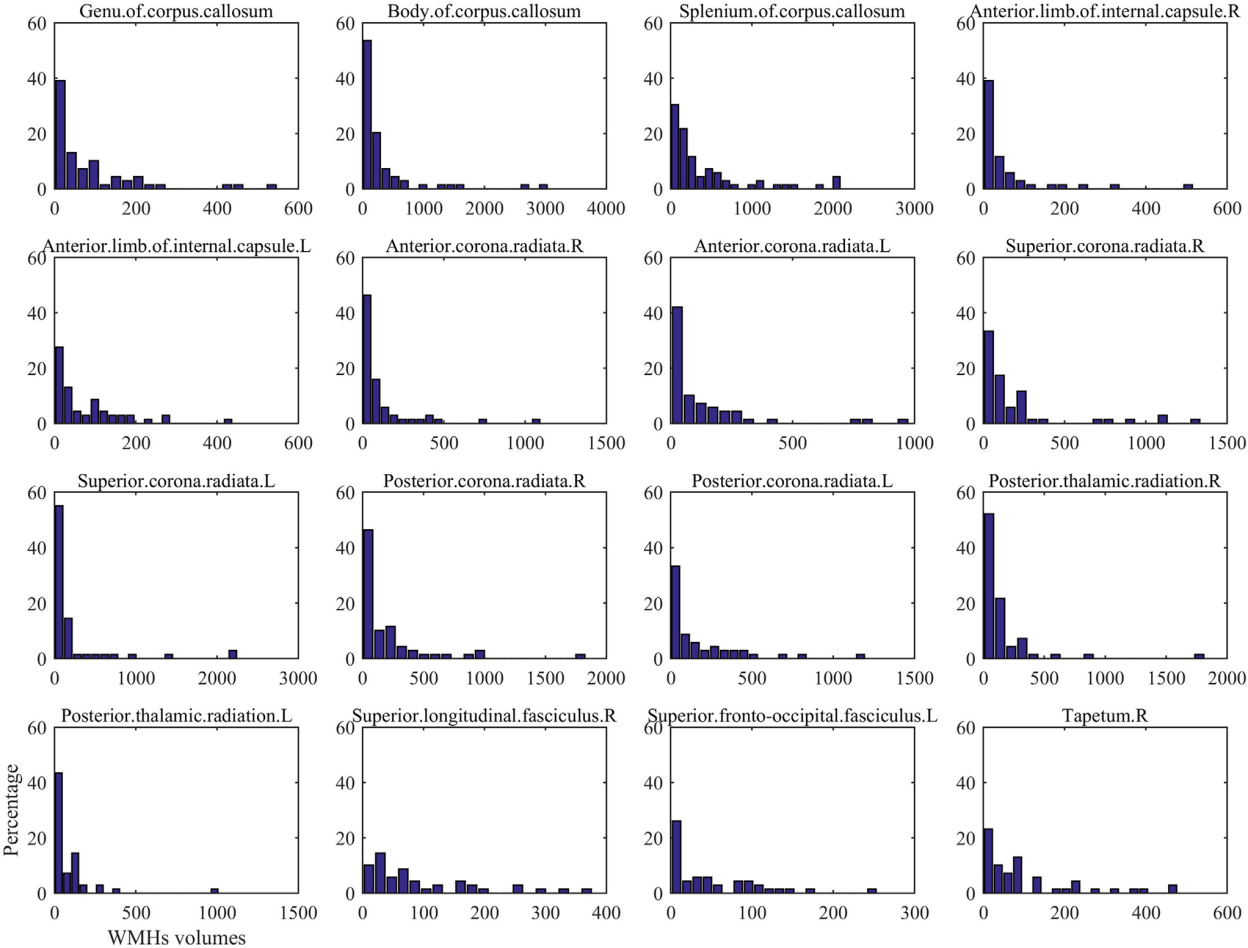
The histogram of White matter hyperintension (WMH) volume distribution in all subjects for each DTI ROI in Figure 1.

The regional distribution of WMHs among all the subjects is given in Figure 1A, which shows most WMHs were located in the periventricular white matter regions (including occipital caps, frontal caps, and lateral bands), and also, some WMHs were scattered in the subcortical white matter regions of frontal, temporal, parietal, and occipital lobes. Since some of the ROIs would not show any WMH in any subject, only the DTI ROIs with more than 60% of subjects showed WMH would be used for further correlation analysis. In this case, 16 DTI ROIs were retained as show in Figure 1B. The histogram of WMH volume distribution in all subjects for each ROI is shown in Figure 2.

**Figure 3:**
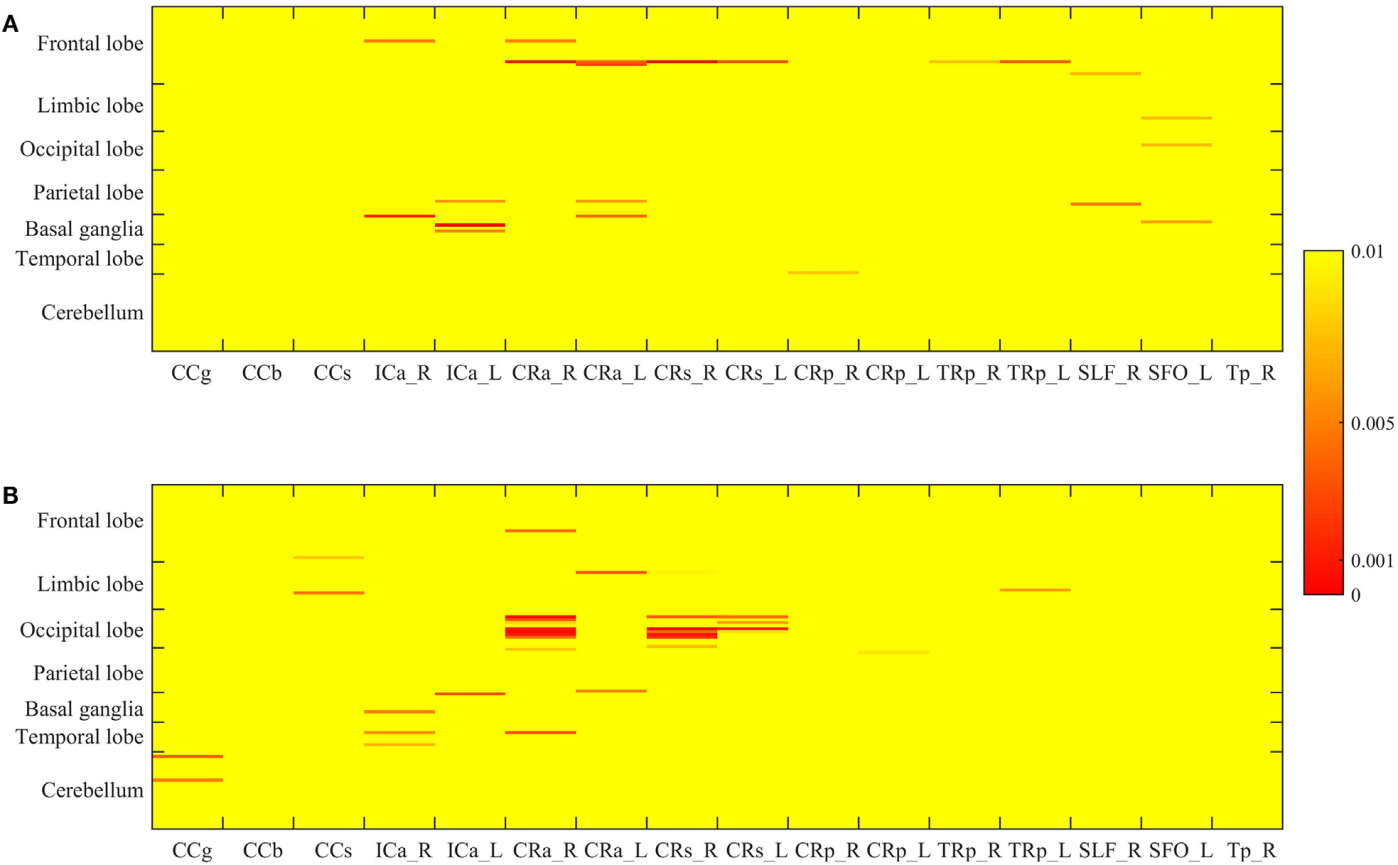
The *p*-value matrix of the correlations of regional WMH volume and cerebral blood flow **(A)**, and those of regional WMH and intrinsic connectivity contrast **(B)**. The color bar presents the *p*-value, with the red showing the *p* < 0.0005. The labels of AAL ROIs of are shown on the left of the matrix, and those of WMH ROIs are shown under the matrix.

**Figure 4:**
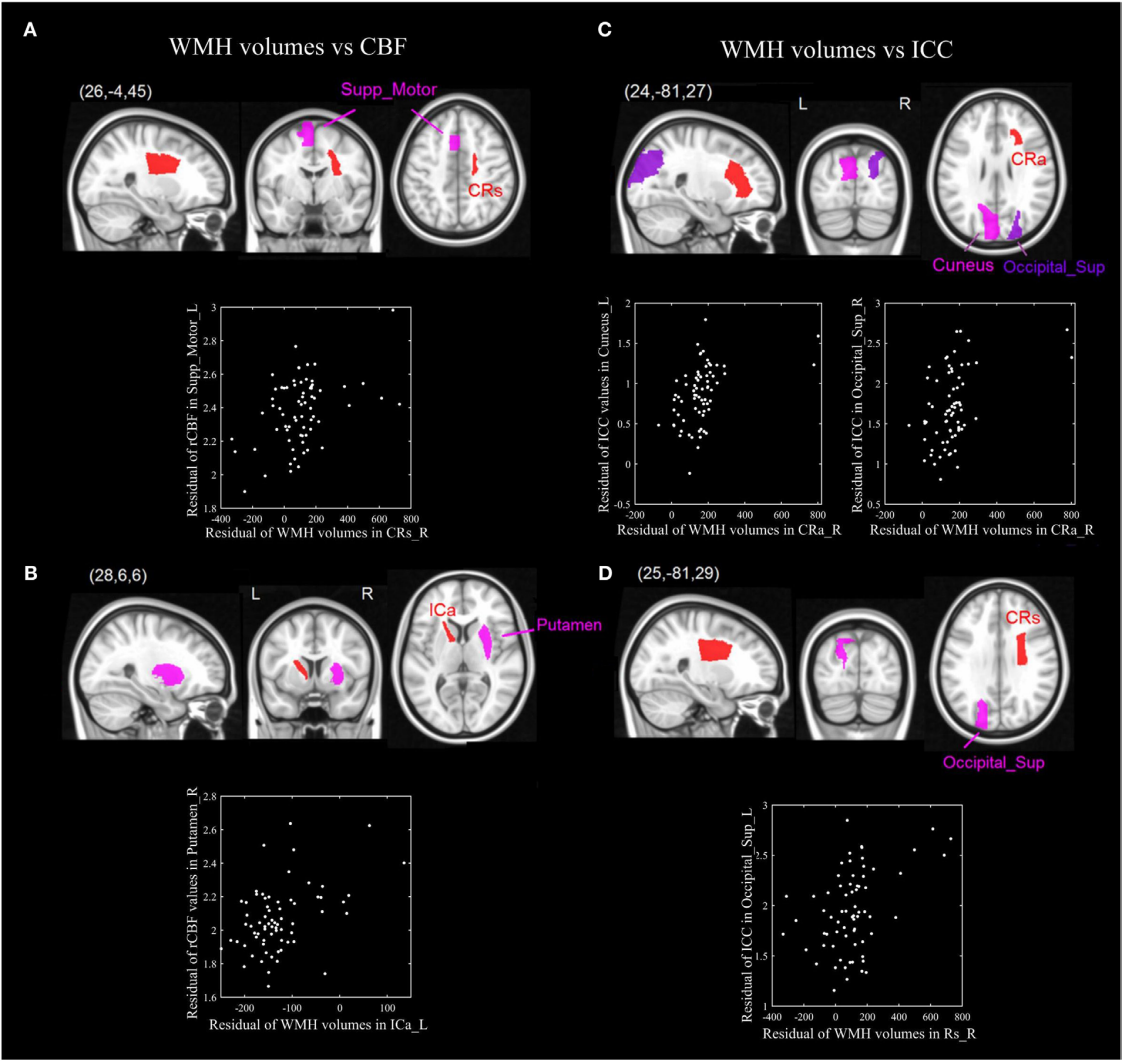
The significant positive correlations of ROIs of WMH volumes and cerebral blood flow (CBF) (left-panel), as well as WMH and intrinsic connectivity contrast (ICC) (right panel). The pairs of ROIs of WMH volumes and CBF which showed significant correlations: the left supplement motor cortex vs. the right superior corona radiata [CRs, **(A)**], and the right putamen vs. the left anterior limb of internal capsule [ICa, **(B)**]. The ROIs of ICC that had significant positive correlations ROIs of WMH volumes were: the left cuneus and the right superior occipital cortex vs. the right anterior corona radiata [CRa, **(C)**], and the superior occipital cortex vs. the right superior corona radiata [CRs, **(D)**].

The *p*-value maps of the correlation matrix of rCBF and WMHs were presented in Figure 3A, and that of ICC and WMHs were in Figure 3B. The correlation results with significant *p*-value (*p* < 0.0005) were shown in red. To better illustrate the significant correlations of regions of WMHs with CBF and ICC, respectively, we presented the regions with significant correlations in red (WMH regions) with magenta (rCBF) or purple (ICC) overlaid on T1W template (Figure 4). The significant correlations of WMH vs. rCBF regions were in the left column, and WMH vs. ICC regions were in the right column. The pairs of regions of rCBF and WMHs, which showed significant positive correlations, included: the left supplement motor cortex vs. the right superior corona radiata [CRs, (A)], and the right putamen vs. the left anterior limb of internal capsule [ICa, (B)]. The regions of ICC that had significant positive correlations regions of WMHs were: the left cuneus and the right superior occipital cortex vs. the right anterior corona radiata [CRa, (C)], and the left superior occipital cortex vs. the right superior corona radiata [CRs, (D)]. The labels of these regions and their *R*- and *p*-values were listed in Table 2 (rCBF vs. WMHs) and Table 3 (ICC vs. WMHs).

**Table 2:** Pairs of ROIs of white matter hyperintensities (WMHs) volume and cerebral blood flow (CBF) which showed significant correlations.

WMHs ROI	CBF ROI	*p*-Value	*r*-Value
Superior corona radiata R	Supp_Motor_Area L	4.47 × 10^−4^	0.42
Anterior limb of internal capsule L	Putamen_R	3.36 × 10^−4^	0.43

**Table 3:** Pairs of ROIs of white matter hyperintensities (WMHs) volume and intrinsic connectivity contrast (ICC) which showed significant correlations.

WMHs ROI	ICC ROI	*p*-Value	*r*-Value
Anterior corona radiata R	Cuneus_L	2.58 × 10^−4^	0.44
Anterior corona radiata R	Occipital_Sup_R	4.24 × 10^−4^	0.42
Superior corona radiata R	Occipital_Sup_L	4.53 × 10^−4^	0.42

## Discussion

This is the first study exhaustively investigating the correlation of regional WMHs with regional CBF and spontaneous brain activities in elderly, using a novel exploratory multimodal MRI analysis approach. Although the regional WMHs had predictable correlations with the total WMHs volume (14), we detected effects of the regional WMHs that could not only be explained by the total WMHs volume alone. The principal findings were that gray matter regions of rCBF and ICC maps located in basal ganglia, parietal, temporal, and occipital cortex, had significant correlations with WMHs volumes of internal/external capsule, and corona radiata.

The rCBF provides the perfusion measurement of the brain, and ICC encodes the voxel-wise functional connectivity information. The converging but differentiable evidences provided by rCBF and ICC are complementary and could form a clearer picture of the functional changes of the brain induced by WMC. Previous literatures have reported the association between WMHs and local CBF (10). The resting-state BOLD signal itself showed reduced physiological noise in WMHs compared to normal aging white matter (22), which may reflect the hemodynamic changes due to the small-vessel disease. The hemodynamic functions of blood pressure and WMHs were associated with medial temporal lobe atrophy in Alzheimer’s disease, which may fill the gap by providing the rationale on how vascular factors could ultimately result in AD (23). The investigation of both CBF and resting-state functional connectivity in our study would provide new insights into the differences and correlations of multiple hemodynamic modalities.

In our study, correlations were found in anatomical region with WMH and functional gray matter. Disregarding the laterality of such correlations, we could look into the anatomical relationships of different regional GM and regions with WMH as illustrated below. (i) The WMH volumes of corona radiata showed a significant positive correlation with the rCBF in the supplement motor area, and ICC of the superior occipital cortex and cuneus (Figures 4A,C,D): The corona radiata contains descending fibers from the motor cortex to the basal ganglia, midbrain motor nuclei and the spinal cord, and ascending fibers from the thalamus to the visual cortex, somatosensory cortex, and the auditory cortex. The associations between the corona radiata and the supplement motor, superior occipital cortex and the cuneus in our results were supported by the anatomical connections. According to Figure 1, (i) we could find that the corona radiata was one of the major areas suffering from WMHs, so the associations of corona radiata with GM cortex may shed light on the mechanism of how WMHs could affect cognitive functions. This result was also consistent with previous studies that the WML volume of superior corona radiata was associated with cognitive speed and flexibility (such as executive performance) (14, 24). (ii) We could also find the rCBF of putamen vs. WMH volume of the anterior limb of ICa (Figure 4B): the ipsilateral anterior limb of ICa is contiguous to putamen, and many fibers of the anterior limb of ICa go through the putamen. In addition, the anterior limb of ICa is an important pathway for transferring information between the thalamus and the cingulate gyrus as well as prefrontal cortex, which has been shown to be significantly associated with memory and executive function (16) in WML patients.

Another interesting finding of current study is that, most rCBF and ICC regions showed significant positive correlations with *contralateral* WMH regions, which indicated rCBF and ICC increased with the increase of contralateral WMH volume (i.e., a more extensive WMC). Literatures suggested that mild and moderate WMH load on cognitive performance is relatively limited, and only severe WML would have clinically relevant effects (25). It has been postulated that there might be a certain cerebral mechanism to counteract the effects of WMHs. Our results supported the functional compensatory hypothesis in aging (26). Many BOLD functional studies reported increased brain activity in aging (27), and evidence indicating that aging will cause over-recruitment of contralateral regions (28). Numerous studies related to aging studies have reported reduced lateralization of many cognitive functions (from various cognitive domains) in elderly, in which elderly subjects tend to recruit bilateral prefrontal cortex in episodic memory retrieval, episodic encoding, working memory and perception tasks. Healthy elderly adults who had high performances tend to reorganize brain functions by over-recruiting the contralateral cortex, while the elderly subjects with low performances recruited a unilateral cortex similar with young subjects, which was ineffective. WML may impair the ability to distribute processing across hemispheres (29), resulting in the cognitive decline in elderly adults. However, previous studies left a gap on how regional WMHs influence the compensation of contralateral GM activities, and our study has potentially built a bridge to link those two above.

There are also some limitations in this study. First, only the WMHs were involved in this study, some of the subjects might also have presence of other cerebral small-vessel disease, such as lacunes, microbleeds, etc., which were not taken into account. It would be very interesting to investigate the relationships between cerebral small-vessel diseases imaging biomarkers and functional connectivity changes in future study. Second, no multiple comparison correction approach was used in this study. In this study, a relatively small threshold *p* < 0.0005, which is similar as the threshold corrected by Bonferroni correction (0.05/116 = 0.00043), was used to reveal the significant relationships. A multiple comparison correction could provide more substantial results in future study with larger sample size. In addition, we speculates that WMH might induce the functional changes of the gray matter activities, further longitudinal study could provide substantial evidences to explore the causality between WMH and gray matter activities changes.

## Summary

To summarize, this study provided a novel angle to investigate the regional effects of WMHs on the gray matter activities independent of total WMH volume in elderly subjects, this provides a new insight to researchers to further understand the mechanism of the cognitive impairment related to WMC. The positive correlations between the regional WMHs volume and the contralateral gray matter activities supported the hypothesis of functional compensation in elderly people.

## Ethics Statement

This study was approved by the Joint Chinese University of Hong Kong-New Territories East Cluster Clinical Research Ethics Committee (CUHK-NTEC CREC) following the ethical standards and procedural requirements described in the Hospital Authority Guide on Research Ethics and the Standard Operating Procedure of the CUHK-NTEC CREC. Formal written consent was obtained from all the participants.

## Author Contributions

LS designed the study and experiments and contributed to drafting the main body of the manuscript. XM contributed to data analysis and manuscript preparation. WL and KL performed data preparation. JA and WC are responsible for data collection. AW and VM managed the subject recruitment and clinical evaluation. DW prepared the study design and data analysis.

## Conflict of Interest Statement

The authors declare that the research was conducted in the absence of any commercial or financial relationships that could be construed as a potential conflict of interest.

## Footnotes

^1^http://www.fil.ion.ucl.ac.uk/spm/software/spm12/.

^2^https://www.nitrc.org/projects/conn/.

^3^http://www.gin.cnrs.fr/AAL?lang=en.

^4^http://www.loni.usc.edu/ICBM/Downloads/Downloads_DTI-81.shtml.
